# Book Reviews in Brief

**Published:** 1914-01-31

**Authors:** 


### Book Reviews in Brief.

Home Health and Domestic Hygiene. By Sir John Collie, M.D., and C. F. Wightman, F.R.C.S. (London: George Gill and Sons. 1913. Pp. 189. Price 9d. net.)

The purchasing power of the sum of ninepence is a problem which has been powerfully exercising the minds of large sections of the community; it is a pleasure to indicate one direction at least in which ninepence may be spent with the full assurance that good value will be obtained. The publishers of this little book on domestic hygiene have treated their customers well both in quality and quantity, and we can without hesitation recommend this manual to all students of hygiene, particularly to health visitors. The names of the authors are sufficient guarantee of the soundness and practical value of the teaching therein expounded, but we have been especially impressed with the excellent selection of the matter. In short, pithy chapters they have enunciated all those little facts and rules which go for the leading of a healthy life and the avoidance of those disastrous fallacies which add so much ill-health and disease among those who cannot afford to be at any disadvantage. The scope of the manual ranges from domestic hygiene to personal hygiene, and includes food, exercise, clothing, infant feeding, and some of the infectious diseases. It is a rare pleasure to welcome such a cheap and praiseworthy production.

Contributions to Practical Medicine. By Sir James Sawyer, M.D., F.R.C.P. Fifth Edition. (Birmingham: Cornish Bros. Pp. 413. Price 5s.)

When a book which is essentially not a "cram" book for students, but a series of clinical studies and essays for practising medical men, reaches a fifth edition, no further proof is wanted as to its own and its author's merits. On previous occasions we have expressed a high opinion of this work, and have seen no reason to modify it. Indeed, as was only to be expected, Sir James Sawyer's clinical wisdom grows riper as the years roll on. It has reached a mellowness which easily accounts for the success of the book, and reflects incidentally lustre on the Birmingham medical school.

Obstetrics for Nurses. By Joseph B. De Lee, M.D. (Philadelphia and London: W. B. Saunders. 1913. Fourth Edition. Pp. 588. Price \$2½ net.)

This book is well produced, well illustrated, and well written. We do not think it is so well suited for the requirements of British nurses as one or two of the best home-made text-books on this subject, and it is by no means cheap; but certainly it is quite sound and unexceptionable. The great detail in which the nursing of premature infants is described is one of the book's really strong points; a weak one, in our opinion, is the statement that syphilis is a disease "which is almost ineradicable and is transmissible even to the third generation."

The Pocket Medical Dictionary. By W. A. Newman Dorland, A.M., M.D. Eighth Edition. (London and Philadelphia: W. B. Saunders. Pp. 677. Price \$1.)

This abbreviation of the same editor's larger work is a most attractive little volume bound in flexible leather. It does not, naturally, afford the encyclopædic completeness of bigger and more expensive medical dictionaries, but it is an uncommonly good dollar's worth. The new edition has been well revised and kept up to date by the inclusion of hundreds of those new terms which are constantly required to express novel conceptions in medicine and the allied sciences. The spelling is American, but English alternatives are also indexed.

